# Recent Aptamer-Based Biosensors for Cd^2+^ Detection

**DOI:** 10.3390/bios13060612

**Published:** 2023-06-02

**Authors:** Zihan Gao, Yin Wang, Haijian Wang, Xiangxiang Li, Youyang Xu, Jieqiong Qiu

**Affiliations:** 1 Zhejiang Provincial Key Laboratory of Silkworm Bioreactor and Biomedicine, College of Life Sciences and Medicine, Zhejiang Sci-Tech University, Hangzhou 310018, China; 2 Hangzhou Alltest Biotech Co., Ltd., Hangzhou 310000, China

**Keywords:** cadmium ions, DNA aptamer, electrochemical, fluorescent, colorimetric

## Abstract

Cd^2+^, a major environmental pollutant, is heavily toxic to human health. Many traditional techniques are high-cost and complicated; thus, developing a simple, sensitive, convenient, and cheap monitoring approach is necessary. The aptamer can be obtained from a novel method called SELEX, which is widely used as a DNA biosensor for its easy acquisition and high affinity of the target, especially for heavy metal ions detection, such as Cd^2+^. In recent years, highly stable Cd^2+^ aptamer oligonucleotides (CAOs) were observed, and electrochemical, fluorescent, and colorimetric biosensors based on aptamers have been designed to monitor Cd^2+^. In addition, the monitoring sensitivity of aptamer-based biosensors is improved with signal amplification mechanisms such as hybridization chain reactions and enzyme-free methods. This paper reviews approaches to building biosensors for inspecting Cd^2+^ by electrochemical, fluorescent, and colorimetric methods. Finally, many practical applications of sensors and their implications for humans and the environment are discussed.

## 1. Introduction

Cadmium ions (Cd^2+^) are deadly poisonous and quickly accumulate in organisms via the food chain. It seriously affects the production and development of plants [1], especially causing great harm to human health. Cardiovascular disease [2], Nephropathy, and Alzheimer’s disease [3] are easily caused by the enrichment of Cd^2+^ in human bodies. Since Cd^2+^ pollution in life mainly comes from the discharge of wastewater from metal smelters, electroplating plants, and mines [4], it is still a problem to be solved. Considering the potent toxicity of Cd^2+^, the content of Cd^2+^ in food and drinking water is strictly regulated. To comply with the Joint FAO/WHO Expert Committee on Food Additives limits, the Cd^2+^ level should not exceed 25 mg/kg body weight per month. Additionally, the Codex Alimentarius Commission regulates the maximum content of Cd^2+^ in polished rice cereals at 0.4 mg/kg [5]. According to the U.S. Environmental Protection Agency (EPA), 44.5 nmol/L (5.0 μg/L) is the maximum limit for Cd^2+^ in drinking water [6].

For monitoring and controlling the Cd^2+^ contamination, analytical methods are necessarily found. A considerable number of traditional methods have been carried out for the measurement of Cd^2+^, for example, inductively coupled plasma–atomic absorption spectrometry (ICP-AES) [7,8], atomic absorption spectrometry (AAS) [9], and inductively coupled plasma-mass spectrometry (ICP-MS) [10]. However, these methods can face challenges when analyzing sophisticated samples due to multiple interfering species. The accuracy of Cd^2+^ detection is easily susceptible to the influence of other metal ion spectra. In the case of monitoring Cd^2+^ in rice, the presence of other metal cations at much higher concentrations can interfere with the accurate detection of Cd^2+^ [11]. Additionally, environmental factors, particularly pH, can further complicate the detection process and limit the sensitivity of the methods [12]. Although the above traditional techniques are feasible, their instruments are expensive and inconvenient. They cannot achieve on-site point-of-care detection and require advanced operational skills and long-term pretreatment. It is these shortcomings that limit the convenient on-site real-time detection of Cd^2+^.

As the unique sequences of DNA can recognize a variety of molecules and ions, DNA is widely used as a biosensor. DNA aptamers are frequently employed to create biosensors to detect a variety of targets, because of their distinctive secondary structures, strong affinity, and selectivity to targets [13]. It provides a means to achieve single-component detection in complex samples. By designing aptamers that specifically recognize the target analyte, biosensors can selectively capture and detect the desired molecule or ion, even in the presence of interfering species. Thus, the construction of electrochemical, fluorescent, and colorimetric biosensors is a quick analytical tool for Cd^2+^ inspection at the molecular level. It has high selectivity and excellent efficiency. Despite the biosensors can accurately identify the target Cd^2+^, its sensitivity is looking forward to improving. Therefore, it is always associated with hybridization chain reaction (HCR) [14], rolling circle amplification (RCA) [15], and entropy-driven catalysis [16] to enhance the detected signal to obtain clear and accurate detection results.

This review first presents the DNA aptamers for Cd^2+^ that are screened by SELEX methods, which obtain high-quality aptamers for relevant targets. Secondly, it summarizes that the DNA biosensors based on aptamers perform a significant role in environmental monitoring, health care, and food inspection. Thirdly, it overviews the application of biosensors for Cd^2+^ detection using electrochemical, fluorescent, and colorimetric methods.

## 2. DNA Aptamers for Cd^2+^

DNA biosensors play an indispensable role in monitoring trace pollutants and studying the interaction between pollutants and DNA, which provides the possible mechanism to explain the toxic action of pollution. Previously, researchers have proposed the use of antibodies [17,18], chelators [19,20,21,22], and DNAzymes [23,24] as recognition factors for Cd^2+^ detection. However, aptamers play a significant role in biosensor procedures compared with such methods due to their reliability and great affinity [10,25,26,27]. Aptamer is a segment of the oligonucleotide sequence, which is typically obtained by in-vitro screening on an oligonucleotide molecular library. In 1990, the traditional SELEX technique was developed by Szostak and Gold groups [28], consisting of selection, distribution, and amplification. It typically takes a long time to obtain a specific candidate aptamer with low hit rates. An improved SELEX method has been established in recent years to attain highly selective aptamers, which can reduce selection time and improve the hit rate.

**Figure 1:**
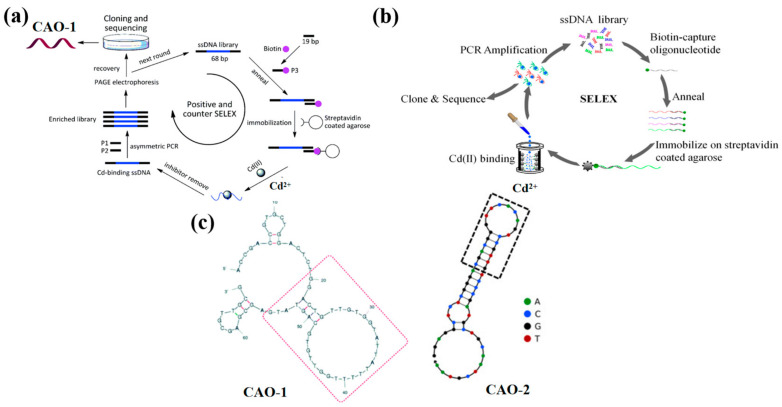
(**a**) The schematic strategy for Cd^2+^ aptamers selection by Wu et al., CAO-1 was obtained [29]; (**b**) The selection protocol of Cd^2+^ aptamers designed by Wang et al., CAO-2 was selected [26]; (**c**) The second structure of CAO-1 and CAO-2.

A new SELEX strategy has been preferred to immobilize the ssDNA libraries instead of the target molecule on the matrix to make the Cd^2+^ aptamer with high affinity (Figure 1a). It received a series of nonrepeat T and G-rich sequences by ten positive tests and one round of adverse selection. Among all sequences, the aptamer CAO-1 (Table 1, Figure 1c) was identified to have the highest binding affinity for Cd^2+^. Their secondary structure shows that the stem-loop structure is the key to the aptamer binding target, which collapses according to the domain of 30 random chains [29]. The dissociation constant (Kd) was used to estimate the aptamer with the highest affinity for Cd(II), which was determined by the relationship between the fluorescence intensity and the concentration of the aptamer using SigmaPlot software with the one-site saturation equation: Y = B_max_X/(Kd + X), where Y represents the fluorescence intensity of Cd^2+^ binding, B_max_ is the maximum Cd^2+^ binding capacity, X is the concentration of the aptamer. The Kd of CAO-1 was calculated to be 34.5 nM. This plan could effectively improve the SELEX process, especially for small molecules such as Cd^2+^, deficient enough sites for immobilization. It will make a potential difference in Cd^2+^ detection and other applications similar to the recovery of contaminated Cd^2+^.

Compared with the above method, an improved SELEX in-vitro selection technique was developed to address the degradability and high cost caused by the long sequences of DNA aptamers, which were designed by the biotin and streptavidin interplay [30,31]. After experimental studies, a shortened aptamer, CAO-2 (Table 1, Figure 1c), was folded into a specific structure to combine Cd^2+^ with high selectivity [26]. As a result, the DNA sequences were released from the ssDNA library, which was captured on the streptavidin column by the target-triggered method (Figure 1b). Also, it efficiently select Cd^2+^ aptamers when the metal ions were free in a liquid solution. The approach revealed that the aptamer could be further curtailed and directly applied as a probe to develop biosensors.

Moreover, an approach selects aptamers as capture probes to identify Cd^2+^ and describes a modified SELEX founded on fixing ssDNA libraries on streptavidin magnetic beads. After nine rounds of screening, four DNA sequences of Cd^2+^ aptamers with the maximum enrichment were obtained. The CAO-3 (Table 1) had the highest affinity for Cd^2+^, with the Kd value being 81.39 μM. The dissociation constant Kd of CAO-3 for Cd^2+^ was calculated the same as CAO-1. Since Cd^2+^ is present in the free state, the strategy can isolate the aptamers more efficiently. The improved SELEX process solves the target selection problem with fewer sites and expands the probe range for Cd^2+^ [32]. To evaluate the specificity of Cd^2+^ aptamers, including CAO-1, the various metal ions, such as Ag^+^, Hg^2+^, Ni^2+^, Ba^2+^, Ca^2+^, Co^2+^, Mn^2+^, Pb^2+^, Zn^2+^, Cu^2+^, and Al^3+^ were tested. The fluorescence response was observed in the presence of Cd^2+^. The sensor system exhibited enhanced selectivity by differentiating Cd^2+^ from other interfering metal ions. Additionally, the fluorescence intensity increased upon the binding of Cd^2+^ to the aptamer, further confirming its selectivity and sensitivity as a sensor for Cd^2+^.

These three SELEX techniques have been successfully used in screening the Cd^2+^ aptamers. Based on DNA aptamers, the sensitive and efficient detection approaches of Cd^2+^ are designed for electrochemical, fluorescent, and colorimetric biosensors. 

## 3. Electrochemical Biosensors

Standard electrochemical detection techniques for Cd^2+^ detection include inductively coupled plasma-mass spectrometry (ICP-MS) [33], inductively coupled plasma emission spectroscopy [34], and electrochemical anodic stripping voltammetry (ASV) [35]. However, their bulky instruments, complex preprocessing, and precise operation often restrict the considerable detection limits and selectivity. Therefore, for the past few years, different nanomaterials have gotten significant attention and have been extensively used in electrochemical biosensors due to their successful synthesis. Owing to their distinctive chemical and physical characteristics, the electrochemical biosensors can detect Cd^2+^ as low as fM. Sensing platforms utilize immobilized Cd^2+^ aptamers on nanomaterial surfaces via catalyzing chemical reactions or intermolecular forces in biosensors to increase electrical signals as sensitizers. This technique enhances biosensor sensitivity [36]. 

### 3.1. Gold Nanoparticles (AuNPs)

In 2017, Liu et al. developed an ultra-sensitive biosensor for detecting Cd^2+^ by modifying glass carbon electrodes (GCE) with AuNPs (gold nanoparticles) and CS (chitosan). The Cd^2+^ aptamer, CAO-4 (Table 1), was neutralized using PDDA (poly- (diallyl dimethyl ammonium chloride)) via electrostatic interactions. Upon the presence of Cd^2+^, the conformation of CAO-4 was altered, resulting in more absorption of [Ru(NH_3_)_6_]^3+^, the electrochemical signal indicator, by the Cd/CAO-4 complex than by PDDA. Differential pulse voltammetry (DPV) revealed an increased peak with increasing Cd^2+^ concentrations. The GCE biosensor demonstrated that it is a promising candidate for monitoring Cd^2+^ levels. It has been successfully applied in tap water samples (Table 2) for Cd^2+^ detection [37].

**Figure 2:**
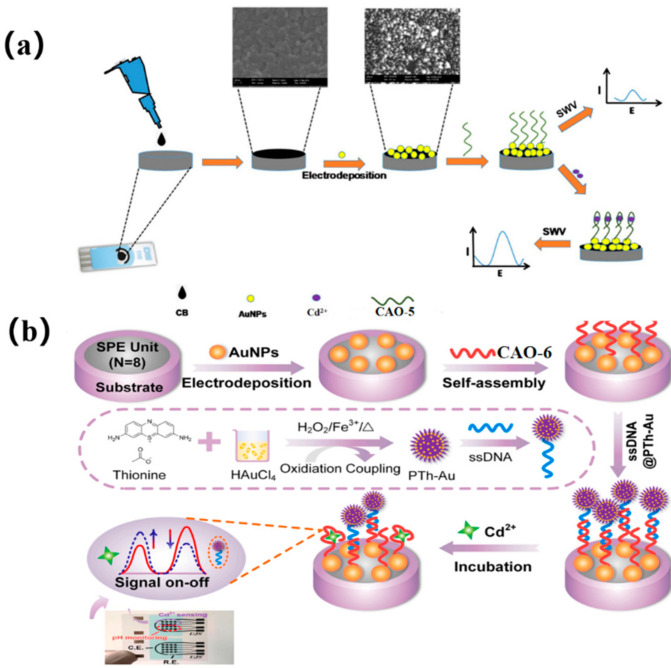
(**a**) diagram of the technical procedure and detection mechanism of the Cd^2+^ biosensor [38]. (**b**) The CAO-6 probes are labeled with 5’−FAM and 3’−Dactyl, designed by Chen et al. [40].

An alternative approach to the previous method was explored using screen-printed carbon electrodes (SPCEs) for manufacturing electrochemical biosensors for Cd^2+^. The SPCEs were shaped using AuNPs and carbon black (CB) (Figure 2a), and a nano-platform was constructed by electrodepositing AuNPs and drop-casting CB via potential cycling from −400 mV to 1100 mV under the scanning rate of 50 mVs^−1^. In the presence of Cd^2+^, CAO-5 (Table 1) immobilized the SPCE-CB-AuNPs, and the conformational change influenced the electrochemical current response. The biosensor exhibited better reproducibility, excellent selectivity (Table 2), and good stability towards Cd^2+^, which had been effectively used to analyze actual water samples [38].

Dual-signal sensing using ratio strategies typically employs either signal-on/off mode or signal-switched mode, with the distinct responses of target analytes depending on their electrochemical properties [39]. The reference signal is kept nearly constant in the signal-switching mode. Thus, Chen et al. also utilized a multipurpose screen-printed electrode (SPE) in synergy with an aptamer couple, creatively using a signal-switching ratio strategy. Onto the SPE, the electrodeposited gold nanoparticles (AuNPs) provided combining points for CAO-6 (Table 1) through Au-S binding, shaping Apt/AuNPs/SPE. In the sensing system, the single-stranded DNA (ssDNA) was designed as a semi-complementary strand of CAO-6, which was bound by polythionate-Au (PTh-Au). This resulted in the formation of ssDNA@PTh-Au, which can self-assemble with Apt/AuNPs/SPE via hybridization. However, ssDNA@PTh-Au was quickly instead of Cd^2+^. Hence, the reference signal (PTh-Au) and the target signal (Cd^2+^) nicely form a radiometric sensing system (Figure 2b) [40]. Compared to the previous two methods, which only showed one signal, the signal-switching mode utilizes both signals to alternate between the target and reference signals. As a result, analytical data, ultimate sensitivity, and accurate reliability can be obtained simultaneously. The biosensor has been successfully used to determine Cd^2+^ in complex biological samples such as clams and mussels, with the low detection limit being 0.006 μgL^−1^ [41].

### 3.2. ZnO Nanocomposites

**Figure 3:**
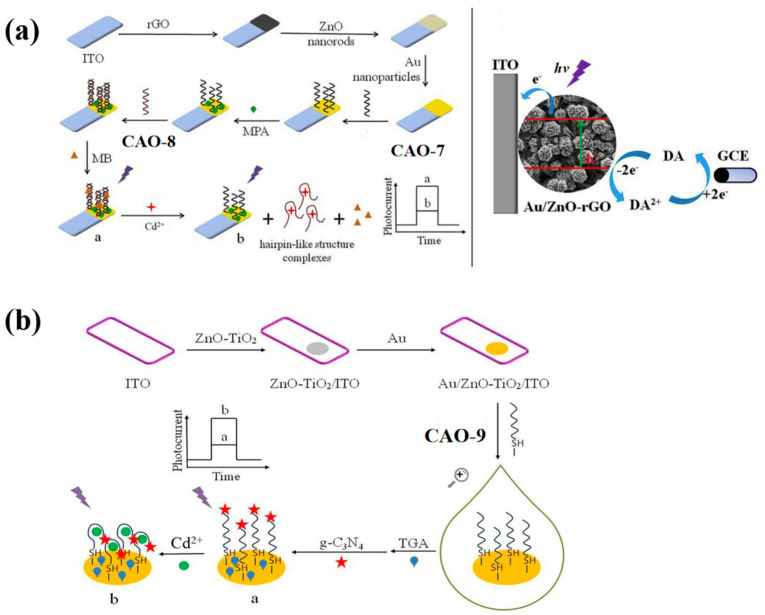
(**a**) Diagram showing PEC’s Cd^2+^ detection strategy and its construction; An electron donor system using four electrodes and DA as the electron donor [43]. (**b**) Detailed schematic of a PEC-adapted sensor for detecting Cd^2+^ [44].

ZnO-rGO nanocomposite was used as the photoactive material to prepare a PEC biosensor. It can specifically recognize the target Cd^2+^ and undergo conformational changes upon identification (Table 2), as illustrated in Figure 3a. Subsequently, AuNPs were incorporated into the ZnO-rGO nanocomposite due to their excellent conductivity and localized surface plasmon resonance. It can enhance the photocurrent signal. CAO-8 (Table 1) will fold into a firm hairpin-like structure because the O and N elements in the G and T bases provide specific sites for Cd^2+^ recognition [29]. To immobilize the SH-shaped aptamer CAO-7, AuNPs acted as anchors. The complementary double-stranded DNA CAO-7 (Table 1) was then modified onto the electrode exterior, pairing with CAO-8. The assembly of methylene blue [42] as a sensitizer in the dsDNA structure enhanced the photocurrent response. To detect the photocurrent, a dual-working electrode system was applied, with the modified electrode and glassy carbon electrode (GCE) as the working electrodes. A cyclic reaction occurred by adding dopamine (DA) to the electrolyte as an electron donor. On the first electrode, DA could be oxidized. On the GCE, it could be reduced. This process enhanced the photocurrent response and improved photocurrent stability, enabling efficient and precise detection of Cd^2+^ [43]. The biosensor had already been utilized for monitoring the water samples and yielding credible results successfully.

In order to simplify the operation, photoelectrochemical (PEC) biosensors have been developed by constructing various sensing interfaces based on the unique identity between the aptamer and target Cd^2+^ [25]. The photosensitive substrates were prepared by modifying electrodes with Au nano-chains (Au NCs) and ZnO-TiO_2_ nanocomposites. The free end of CAO-9 (Table 1) was covalently bonded to g-C_3_N_4_ (graphite-like carbon nitride) to amplify the detection signal. The specific photoelectric property of g-C_3_N_4_ was exhibited with visible light and ultraviolet irradiation (Figure 3b) [44]. Compared to the previous method, this approach produced more sensitive and linear photocurrent signals proportional to the Cd^2+^ concentration for precise quantitative analysis of the target. The signal amplification strategy employed in this method is more straightforward, yet it still achieves high sensitivity and selectivity (Table 2).

### 3.3. Other Materials

**Figure 4:**
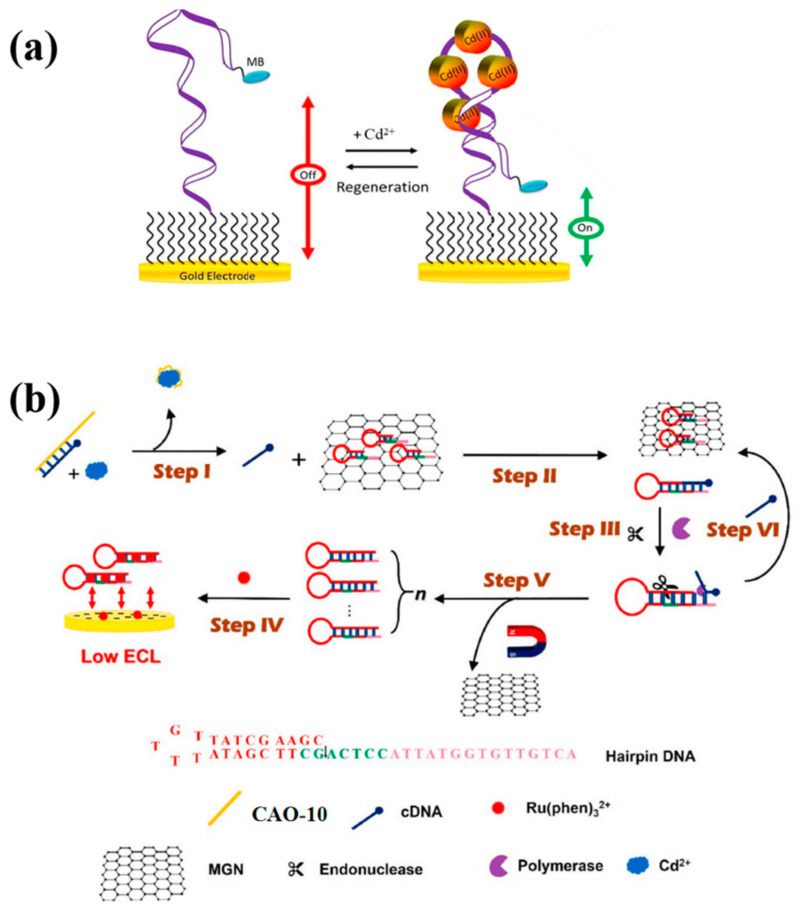
(**a**) The diagram illustrates the construction of “signal-on” E-AB biosensors that are utilized for monitoring Cd^2+^ [25]; (**b**) The schematic diagram was developed for Cd^2+^ detection by Xu et al. [45].

This study implemented the use of the same aptamer CAO-5 mentioned in the previous method to develop a reagent-free and reusable electrochemical biosensor. An E-AB (electrochemical aptamer-based) biosensor was used to detect Cd^2+^. CAO-5 was labeled with methylene blue [42]. When Cd^2+^ is absent, the superficial immobilized aptamer probe was locally folded (Figure 4a). Upon binding to Cd^2+^, the probe CAO-5 changed its confirmation and adaptability, increasing the MB signal [25]. The “signal-on” strategy was used, which is generally more advantageous than “signal-off” in developing biosensors. Parameters that influence the monitoring of Cd^2+^ were systematically optimized in this reagent-free and reusable electrochemical biosensor. Such biosensors have shown high selectivity in complex samples and can be applied directly. Furthermore, immediate results can be obtained through this method, which is also renewable and reusable. It is possible to use it in the measurement of Cd^2+^ in biological and environmental materials in the future with additional improvements.

Recently, researchers carefully created a hairpin DNA that was used in strand displacement amplification (SDA) and structured an electronic biosensor based on magnetic graphene oxide nanosheets (MGN) (Figure 4b). The MGN restricted previously designed hairpin DNA because of the lack of Cd^2+^. It releases cDNA via combination with CAO-10 (Table 1), removing the hairpin DNA on MGN surfaces in the presence of Cd^2+^. In the solution, the electrochemiluminescence (ECL) probe Ru(phen)_3_^2+^ readily diffuses and efficiently reaches the electrode surface, causing an enhanced ECL signal. In this case, SDA amplification is induced to excite and generate a large amount of dsDNA, trapping Ru(phen)_3_^2+^ in its groove. The implanted ECL probe connecting the electrode surface cannot produce an ECL signal. Consequently, the concentration of Cd^2+^ could be detected based on the decay of the detection signal [45]. This technique greatly simplifies the performance procedure. Furthermore, it is appropriate to be utilized in the complicated matrix.

## 4. Fluorescent Biosensors

Traditional methods for detecting Cd^2+^ using fluorescence include a Cyclen-based Fluorescent Chemosensor [46] and a radiometric fluorescent peptide sensor [47]. However, these techniques have drawbacks such as complicated operation, low monitoring sensitivity, and consuming time [48,49,50]. In contrast, fluorescent biosensors based on aptamers offer high sensitivity, precision, and a low detection limit. Whether using label-free/labeled methods or signal amplification strategies, fluorescent biosensors have the potential to replace traditional methods for Cd^2+^ detection [51,52]. 

### 4.1. Label-Free

**Figure 5:**
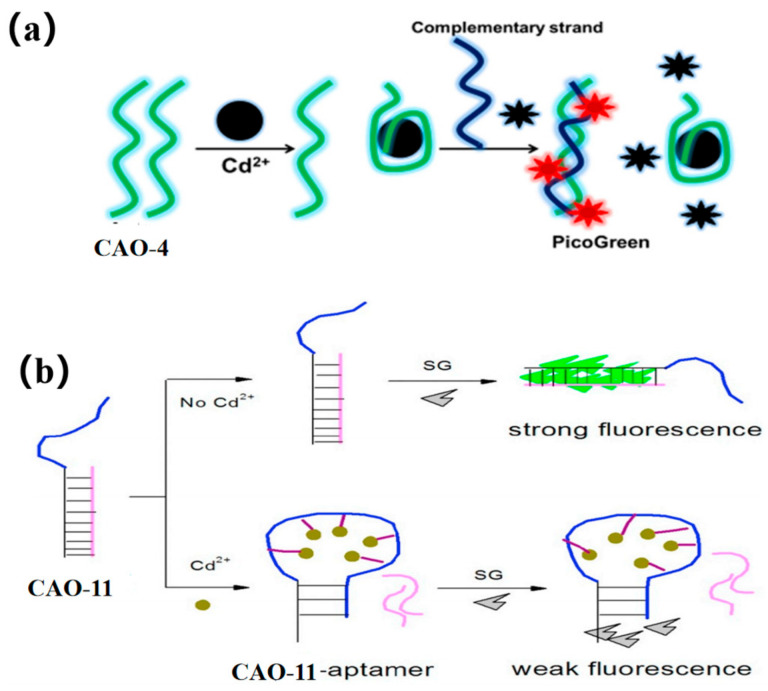
(**a**) PicoGreen (PG) used for Cd^2+^ detection [53]; (**b**) SYBR green applied for the Cd^2+^ determination [55].

PicoGreen (PG) is an asymmetric anthocyanin dye, which cannot fluoresce freely. However, when it is bound to CAO-4 (Table 1) by hybridization with its complementary strand, its fluorescence is increased by more than 1000-fold (Figure 5a). Additionally, it does not exhibit fluorescence when bound to CAO-4 as single-stranded DNA [15]. Thus, PG can be used as a dye that enhances fluorescence based on such a mechanism. By combining an aptamer sensor with this dye [42], an easy, sensitive, and versatile fluorescence sensing plan for Cd^2+^ detection can be developed [53]. This method demonstrated greater sensitivity to the matrix effect when compared to atomic absorption spectrometry [54].

SYBR green is an intercalator dye, which can combine with the minor groove of double-stranded DNA. CAO-11 (Table 1) can hybridize to form a DNA duplex with its complementary strand. After adding SG, the intensity of the fluorescence signal increases (Figure 5b). When Cd^2+^ is present, CAO-11 can bind to it, and DNA hybridization is disrupted [55]. The fluorescent biosensor does not require target preconcentration or sample treatment and exhibits a higher degree of selectivity.

### 4.2. Labeled Method

In 2017, a susceptible and selective Cd^2+^ detection strategy was designed by Zhu et al. The strategy involved the utilization of a multifunctional probe, which was individually labeled and consisted of a specific Cd^2+^ aptamer, CAO-12 (Table 1). The aptamer was responsible for recognizing Cd^2+^ and acted as a signal reporter. Upon interaction with Cd^2+^, the CAO-12 underwent a conformational transition from a free sequence to a hairpin structure. Due to photoinduced electron transfer, the conformational change caused fluorescence quenching. As the concentration of Cd^2+^ increased, the fluorescence response was enhanced, leading to the successful employment of a novel fluorescence approach for monitoring Cd^2+^ [27]. Compared to traditional techniques such as inductively coupled plasma mass spectrometry and inductively coupled plasma atomic emission spectrometry [56], this biosensor only required the utilization of the aptamer probe. The strategy did not require complex processes or sophisticated apparatus. However, further improvements are still needed to enhance its detection limit.

**Figure 6:**
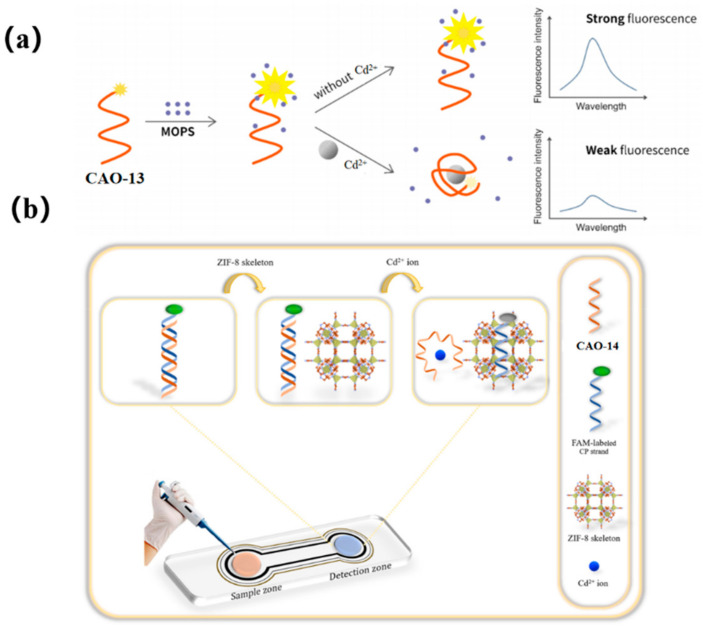
(**a**) A schematic illustrating a simple and ultra-efficient sensor for measuring Cd^2+^, which was developed by Liu et al. [57]; (**b**) Schematic representation of the project biosensor for sensitive detecting of Cd^2+^ [6].

To achieve more accurate and efficient detection of Cd^2+^, a later study found that 3−(N-morpholino) propane sulfonic acid (MOPS), a widely used buffer, could significantly enhance the fluorescent intensity of FAM-labeled CAO-13. Upon adding Cd^2+^, the fluorescence changes and remains stable for at least an hour before decreasing proportionally with the increasing concentration of Cd^2+^. This development led to the creation of a simple adaptive sensor for the instantaneous determination of Cd^2+^, as illustrated in Figure 6a [57]. MOPS was found to possess fluorescence amplification properties with the FAM-aptamer CAO-13, which is uncommon among other commonly used buffers. Despite being sensitive to pH, MOPS could still be practically applied for real-time monitoring of Cd^2+^ due to its easy handling, high performance, and exceptional efficiency as a biosensor.

The latest report introduces an innovative monitoring array for detecting ultra-low levels of Cd^2+^ by utilizing the excellent selectivity and sensitivity of aptamers as sensing probes and the ability of ZIF-8 (zeolitic imidazolate framework-8) to quench fluorescence. To develop a portable spot monitoring technique, the biosensor was prepared utilizing a paper-supported substrate, as shown in Figure 6b. The detection strategy exploited the specific combination of CAO-14 (Table 1) with Cd^2+^ and the distinct affinities between single and double DNA chains for sorption on the ZIF-8 framework. When Cd^2+^ was absent, a CP-CAO-14 rigid duplex was formed through the hybridization between CAO-14 and its complementary (CP) strand, which was labeled with FAM. Adding the CP-CAO-14 complex onto the monitoring area resulted in a negligible trend for sorption by the exterior of the quencher ZIF-8, which led to a notable fluorescence signal. Upon forming the CAO-14-Cd^2+^ complex, the cross of the CP strand and the CAO-14 would be obstructed. Consequently, the ZIF-8 appearance adsorbed the CP strand, quenching the FAM fluorescence. Compared to previous studies on fluorescence biosensors, this approach designed a paper-supported sensing array, which was successfully applied in real complex food samples, including water, rice, and potatoes [6].

### 4.3. Signal Amplification

**Figure 7:**
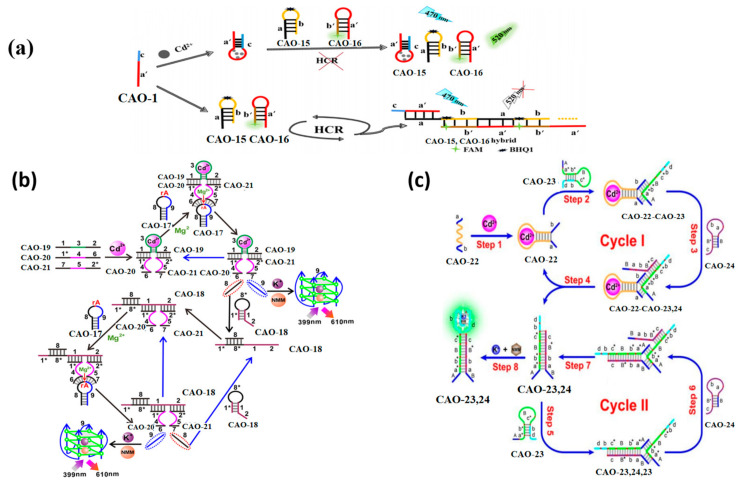
(**a**) Principles of the biosensor detection of Cd^2+^ [58]; (**b**) Illustration of a Cd^2+^ biosensor based on DNAzyme-dependent Mg^2+^ [59]. (**c**) Diagrammatic sketch of monitoring Cd^2+^ by toehold combination and branch migration. [60]. *: was used to distinguish the different domain of each DNA sequence.

A biosensor for Cd^2+^ detection was designed using Cd^2+^ aptamers and a hybridization chain reaction (HCR). The operational principles of the biosensor are illustrated in Figure 7a. The biosensor was based on three oligonucleotide probes, namely CAO-1, CAO-15, and CAO-16 (Table 1). CAO-1 was utilized as a promoter for HCR and a Cd^2+^−specific aptamer, while CAO-15 and CAO-16 were subjected to heating and cooling to form a hairpin structure, respectively. BHQ1 was used to label CAO-15, whereas 5-carboxyfluorescein (FAM), which can emit fluorescence, was used to label CAO-16 [58]. In the absence of Cd^2+^, a pulse on the HCR by CAO-1 will form long-nicked dsDNA structures. However, fluorescence quenching is achieved by binding Cd^2+^ to CAO-1, which prevents HCR from regaining fluorescence. The limit of detection is 0.36 nM, so this can further improve sensitivity in Cd^2+^ detection (Table 2).

This approach utilizes toehold-mediated strand displacement with the aid of Mg^2+^−dependent DNAzyme to develop a versatile Cd^2+^ sensor, as demonstrated in Figure 7b. Domain 3 of CAO-19 (Table 1) is a specific aptamer sequence that can combine with Cd^2^. The split sequences of the DNAzyme consist of domain 4 of CAO-20 (Table 1) and domain 5 of CAO-21 (Table 1). With Cd^2+^, domains 1 and 2 were led to be short enough distance via the interplay between CAO-19 and Cd^2+^ and DNA. Then field 1* of CAO-20 and field 2* of CAO-21 could be hybridized, respectively. CAO-19-CAO-20-CAO-21, a synergistically stabilized construction, produced an active Mg^2+^−dependent DNAzyme that leads the hairpin substrate strand (CAO-17) (Table 1) to be catalyzed to make the cleavage reaction. The trigger in CAO-17 can turn on CAO-18, another hairpin probe enabling the cyclic signal intensification procedure to be activated [59]. The fluorochrome produces many G-quadruplex DNAzyme structures, creating a highly fluorescent response upon incubation with N-methyl mesoporphyrin IX (NMM). The experiment utilizes OR logic gates, allowing the logic system to conduct the function commendably, even with the complicated specimens. This approach is simple to operate, enzyme and label-free, and capable of amplifying universal signals.

Pan et al. designed a more sensitive approach for detecting Cd^2+^ utilizing an enzyme-free DNA circuit according to branch migration and hairpin probe-mediated toehold binding. The identity probe in the DNA circuit is CAO-22 (Table 1), and the reporter gene is a G-quadruplex, making it more convenient to achieve the goal. Figure 7c illustrates the biosensor’s principle for enzyme-free monitoring of Cd^2+^. When Cd^2+^ is present, the structural domains a and b within CAO-22 are nearby, allowing the fragment of CAO-22 to be used as a trigger fragment for subsequent strand substitution reactions with CAO-23 and CAO-24. It forms a G-quadruplex, producing a highly fluorescent response upon incubation with NMM. This method is more sensitive to detecting Cd^2+^ at ultra-low concentrations (Table 2) [60].

## 5. Colorimetric Biosensors

Recently, colorimetric biosensors based on metallic nanoparticle (NP) assays have garnered increasing interest owing to their interparticle distance-dependent optical properties and partial solid surface plasmon resonance absorption [61,62,63,64]. Colorimetric biosensors offer several advantages, including easy measurement, rapidity, high sensitivity, simplicity, and cost-effectiveness [65,66].

### 5.1. Gold Nanoparticles

**Figure 8:**
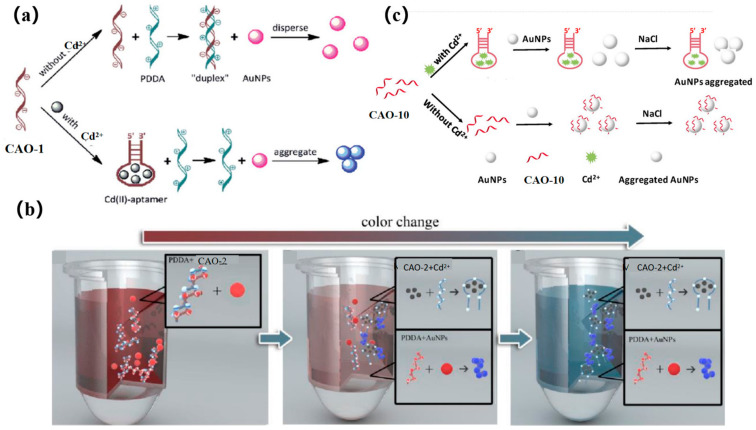
(**a**) A diagram of the colorimetric detection of Cd^2+^ using AuNP aggregation mediated by cationic polymers using CAO-1 aptamers for recognition [29]; (**b**) Colorimetric method illustrated for Cd^2+^ detection [67]; (**c**) Colorimetric principle was designed for Cd^2+^ detection based on the functional AuNPs [10].

The accumulation of AuNPs is mediated via a cationic polymer to produce a colorimetric signal. In early research, CAO-1 (Table 1) was considered the Cd^2+^−based recognition element for cationic polymer (AuNPs) aggregation for colorimetric detection (Figure 8a) [29]. In the absence of Cd^2+^, the CAO-1 is free and can form a “duplex” structure by hybridizing with PDDA, which prevents AuNPs from aggregating due to the lack of PDDA. In the presence of Cd^2+^, CAO-1 is consumed, leading to a significant color change from red to blue due to the accumulation between PDDA and AuNPs. This method can accurately detect Cd^2+^ in an aqueous solution. If CAO-1′s selectivity can be improved, the method will be valuable in applications such as Cd^2+^ detection or remediation of contaminated Cd^2+^.

A more recent and innovative approach for testing Cd^2+^ involves a simple combination of CAO-2 and AuNPs by a colorimetric mobile phone readout. The technique utilizes a competitive binding assay between CAO-2, PDDA, and Cd^2+^. AuNPs act as signal reporters, and the color changes from red to blue, increasing the Cd^2+^ concentration, and resulting in free PDDA availability. Additionally, mobile phones equipped with ColorAssist applications can capture the detection results (Figure 8b). The detector can observe the image’s red (R) values with different signal intensities in response to different concentrations of Cd^2+^. This technique has already been employed for detecting and quantifying Cd^2+^ in rice and drinking water. As a result, low-cost and convenient colorimetry demonstrates its potential for practical use in quantitative and visual monitoring with a mobile phone [67].

Recently, a colorimetric approach for monitoring Cd^2+^ based on CAO-10 (Table 1) and the functionalized AuNPs has been developed for unique recognition, and it is more efficient in terms of colorimetric smartphone readings. In high-salt solutions, AuNPs tend to aggregate due to the screening of electrostatic repulsion. However, CAO-10 can prevent aggregation and enhance the stability of AuNPs. When Cd^2+^ exists, CAO-10could react with Cd^2+^, which results in a reduction of the free CAO-10. It leads to a color alternation of the solution and decreased stability of AuNPs (Figure 8c) [10]. The detection limit is 1.12 g/L, less than the USEPA’s permitted level for drinking water (5 μg/L) [68]. Compared to the previously mentioned method using a smartphone, this colorimetric system can be analyzed and captured by a smartphone within 10 min, and it can also achieve quantitative and in-situ monitoring of Cd^2+^ (Table 2).

### 5.2. Other Nanoparticles

**Figure 9:**
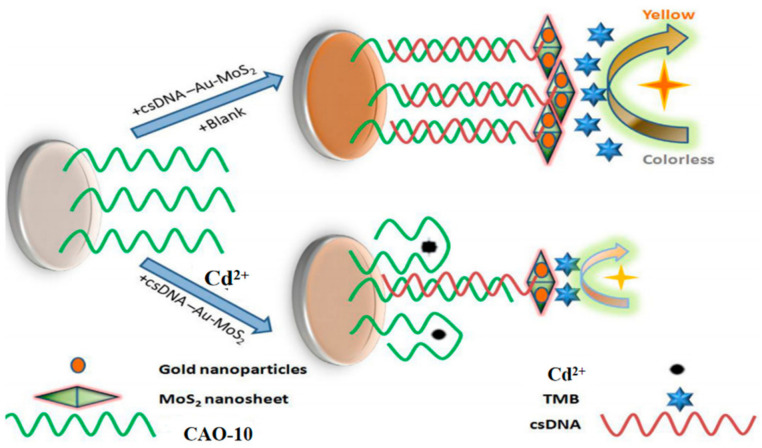
The principle of Cd^2+^ detection by this assay [69].

A new approach based on gold nanoparticle-modified molybdenum disulfide nanocomposites has been developed for monitor Cd^2+^. In this approach, the biotinylated Cd^2+^ aptamer CAO-10 (Table 1) is immobilized on a microplate through biotin-affinity binding. Additionally, a signal probe called csDNA-Au-MoS_2_ is made up of complementary strands of CAO-10 adsorbed onto the gold nanoparticle-modified molybdenum disulfide composite. When target Cd^2+^ competes with the csDNA-Au-MoS_2_ for binding to CAO-10, a color change is observed upon the addition of a chromogenic substrate (Figure 9) [69]. It was applied for Cd^2+^ detection in white wine samples, demonstrating its practical applicability. This approach is relatively complex to operate but has relatively high sensitivity (Table 2).

This colorimetric method for determining Cd^2+^ employed Mn_3_O_4_ nanoparticles (NPs) with oxidase-mimicking function, which could be regulated by oligonucleotides. The chromogenic agent tetramethylbenzidine (TMB) can be oxidated by the catalyst of Mn_3_O_4_ NPs, resulting in a yellow product in acidic solutions. When CAO-25 (Table 1) is absorbed onto the surface of Mn_3_O_4_ NPs, TMB is temporarily inhibited from oxidizing, and the solution appears light green due to decreased absorbance. However, when Cd^2+^ is present, the inhibition is canceled, and thymine bases in CAO-25 bind to Cd^2+^, changing the solution’s color from light green to yellow. This method provides a more rapid and accurate determination of Cd^2+^, and it is a simple, evident, and selective process [70].

### 5.3. G-4/Hemin-Assisted Method

This assay exploited a label-free aptamer biosensor for Cd^2+^ determination using an aptamer specific to G-quadruplex-Cd^2+^ (GCDSA). GCDSA was designed by combining a G-rich sequence and an essential structural domain of CAO-26 (Table 1) to recognize Cd^2+^ and act as a signal DNAzyme specifically. When no Cd^2+^ exists, GCDSA remains mostly a random coil sequence. However, upon adding Cd^2+^, G-quadruplex formation can be induced in GCDSA, which can combine with hemin to act as peroxidase-like DNAzymes. It increases the signal of the sensing design, and the Cd^2+^ concentration can be determined by observing the absorbance. This method avoids using labeled oligonucleotides, makes the experimental procedure simple and convenient, allows for direct quantitative sample analysis, and has an excellent dynamic range. It displays two segments with a broad linear range, allowing Cd^2+^ to be detected at concentrations as low as 0.15 nM [71].

**Figure 10:**
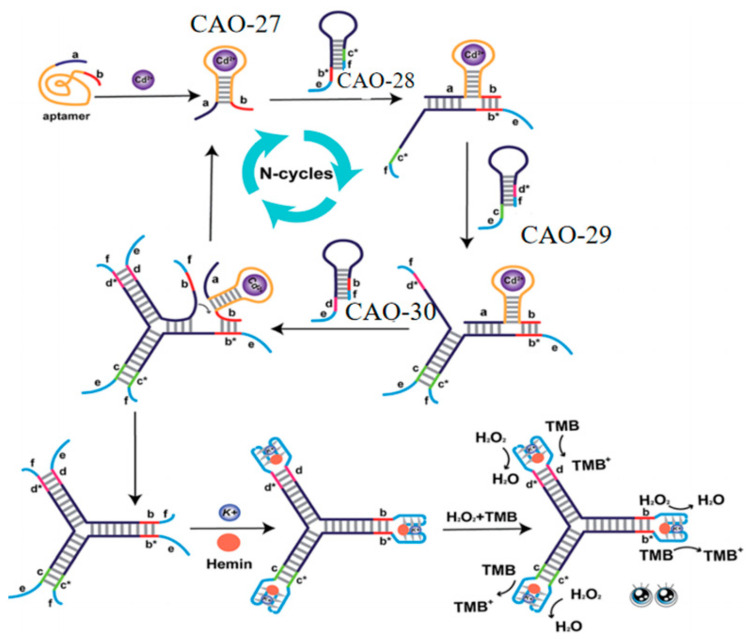
DNA Y junctions containing the G-quadruplex act as the label-free signal reporters to monitor Cd^2+^ [72].

Lastly, to further improve sensitivity and convenience, a label-free and enzyme-free three-way ligation sensing system was proposed for Cd^2+^ detection by G-quadruplex/heme DNAzyme. Three hairpin structures (CAO-28, CAO-29, CAO-30) (Table 1) were designed to build three-way G-quadruplex linkages as the building blocks. When Cd^2+^ is present, the binding of Cd^2+^ to CAO-27 (Table 1) results in the branching migration of the hairpin and the formation of an unstable complex. The Cd^2+^−CAO-27 separates from the complex and becomes a catalyst that allows additional branching connections to be hybridized. It leads to the formation of G-quadruplexes. Upon adding heme, many intact G-quadruplex/heme DNAzymes can be produced, enhancing the color changes observable to the naked eye (Figure 10). This biosensor has excellent selectivity and sensitivity for Cd^2+^ monitoring. It is also simple to operate without washing, modifying, or labeling. It is practical for the routine detection of Cd^2+^ [72].

**Table 2:** Comparison of the biosensors for Cd^2+^ monitoring based on electrochemical, fluorescent, and colorimetric methods in sensitivity and detection range.

Method	No.	LOD	Detection Range	Application	Ref.
**Electrochemical detection**	CAO-4	0.04995 pM	0.001~100 nM	Tap water	[37]
	CAO-5	0.14 ppb	1~50 ppb	Drinking water	[38]
	CAO-6	7 × 10^−4^ mg/L	2 × 10^−3^~8 × 10^−1^ mg/L	Genuine mussels	[40]
	CAO-7	1.8 × 10^−12^ mol/L	5.0 × 10^−12^~2.0 × 10^−8^ mol/L	Lake water	[43]
	CAO-9	1.1 × 10^−11^ mol/L	3.0 × 10^−11^~4.0 × 10^−8^ mol/L	Lake water	[44]
	CAO-5	92 nM	250~1 μM	Tap water	[25]
	CAO-10	1.1 × 10^−4^ ppb	3.0 × 10^−2^~4.0 × 10^5^ ppb	Traditional medicine	[45]
**Fluorescence detection**	CAO-4	0.038 ng/mL	0.10~100 µg/mL	Drinking water	[53]
	CAO-11	0.34 μg/L	1.12~224.82 μg/L	Drinking water	[55]
	CAO-12	2.15 nM	7.19 nM~5.0 μM	Drinking water	[27]
	CAO-13	1.92 ng/mL	5~140 ng/mL	Drinking water	[57]
	CAO-14	0.076 pM	0.1~120 pM	Milk, coffee, and human blood serum	[6]
	CAO-1	0.36 nM	0~10 nM	Pond water	[58]
	CAO-17	2.5 pM	0~10 μM	Rice	[59]
	CAO-22	5 pM	10 pM~100 μM	Human urine	[60]
**Naked** **eye observation**	CAO-1	4.6 nM	1~50 nM	Drinking water	[29]
	CAO-2	1 ng/mL	1~400 ng/mL	Rice and drinking water	[67]
	CAO-10	1.12 μg/L	2~20 μg/L	Tap and drinking water	[10]
	CAO-10	0.7 ng/mL	1~500 ng/mL	White wine	[69]
	CAO-25	2.4 μg/L	5~100 μg/mL	Tap, river, lake, wastewater	[70]
	CAO-26	0.15 nM	33.72~112.41 μg/L	Tap and pond water	[71]
	CAO-27	10 pM	10 pM~1 μM	Tap, river, lake, wastewater	[72]

## 6. Conclusions

This article provides an overview of SELEX, a new technology used to extract DNA aptamers that can be utilized as electrochemical, fluorescent, and colorimetric biosensors. These biosensors have the potential to detect harmful pollutants in water and food, particularly Cd^2+^. DNA biosensors offer several advantages over traditional methods, such as inductively coupled plasma atomic emission spectrometry and atomic absorption spectrometry. One of the main advantages is their simpler pre-preparation and operation. DNA biosensors involve the design and synthesis of specific DNA probes, which can be easily prepared in the laboratory setting. Additionally, the detection process in DNA biosensors is relatively straightforward and does not require complex sample preparation steps. However, traditional methods often rely on expensive and bulky equipment, such as ICP-AES or AAS instruments, which may not be easily accessible or affordable for every laboratory or field application. In contrast, DNA biosensors can be designed to work with more straightforward and compact instrumentation, making them more cost-effective and portable.

The biosensors designed using specific and high-affinity Cd^2+^ aptamers have demonstrated excellent sensor behavior, surpassing other methods regarding detection limits and detection range comparisons. These biosensors exhibit exceptional selectivity and sensitivity due to efficient signal amplification strategies employed in their design. However, biosensors based on the Cd^2+^ aptamer design are still at an early stage of development. The specificity and productivity of the SELEX approach for Cd^2+^ aptamers can be challenging, as it is a time-consuming process that may yield a limited number of aptamers. This limitation can hinder aptamer-based biosensors’ affinity and binding efficiency, ultimately impacting their sensitivity. Moreover, when applying these biosensors to natural samples, additional factors must be considered. Environmental factors, such as proteins, DNA sequences, and enzymes in the sample, can interact and bind to Cd^2+^ aptamers, leading to interference and potentially restricting the detection of Cd^2+^. These factors can affect the accuracy and reliability of biosensor results obtained from laboratory experiments. Therefore, further research and development efforts are necessary to overcome these challenges and achieve practical monitoring of Cd^2+^ using biosensors.

DNA aptamers offer a promising approach for detecting Cd^2+^ in the environment and ensuring food safety. Nonetheless, more work is required to overcome the current challenges and optimize the potential of these biosensors. Indeed, real-time detection is a valuable function increasingly achieved by biosensors based on aptamers. These biosensors offer advantages such as high sensitivity, selectivity, and the ability to recognize specific targets. It is anticipated that aptamer-based biosensors will continue to evolve and enable real-time monitoring and in vivo detection with high sensitivity. Overall, the future of aptamer-based biosensors looks promising, and their continued development and application will likely pave the way for real-time monitoring and in vivo detection with high sensitivity across various fields and applications.

## Figures and Tables

**Table 1:** DNA sequences of the mentioned probe, and the Cd^2+^ aptamer sequences are underlined.

Probe	Sequences of Nucleotide
CAO-1	GGACTGTTGTGGTATTATTTTTGGTTGTGC
CAO-2	CTCAGGACGACGGGTTCACAGTCCGTTGTC
CAO-3	TTAAGTTTGGGACAAGTTTAGCCTTTGCGTGGATGTGGCT
CAO-4	ACCGACCGTGCTGGACTCTGGACTGTTGTGGTATTATTTTTGGTTGTGCAGTA TGAGCGAGCGTTGCG
CAO-5	SH−(CH_2_)_6_GGACTGTTGTGGTATTATTTTTGGTTGTGCAGTATG
CAO-6	SH-TTTTCGACGGGTTCACAGTCCGTTG
CAO-7	SH-CATACTGCACAACCAAAAATAATACCACAACAGTCC
CAO-8	GGACTGTTGTGGTATTATTTTTGGTTGTGCAGTATG
CAO-9	SH−GGACTGTTGTGGTATTATTTTTGGTTGTGCAGTATG-NH_2_
CAO-10	ACCGACCGTGCTGGACTCTGGACTGTTGTGGTATTATTTTTGGTTGTGCAGTATG AGCGAGCGTTGCG
CAO-11	GGGAGGGAACTGTTGTGGTATTATTTTTGGTTGTGCAGTAGGGCGGG
CAO-12	GGGGACTGTTGTGGTATTATTTTTGGTTGTGCAGT
CAO-13	GGACTGTTGTGGTATTATTTTTGGTTGTGCAGTCC
CAO-14	ACTGTTGTGGTATTATTTTTGGTTGTGCAGTA
CAO-15	CCAAAAATAATACCACAACAGTCCCAAAGTGGACTGTTGTGGTATTAT
CAO-16	GGACTGTTGTGGTATTATTTTTGGATAATACCACAACAGTCCACTTTG
CAO-17	CCCAACCAAATGACGATrAGGGTAGGGCGGGTTGGG
CAO-18	GACGAGTCGCATATCGTCATTTGGTTGGGATGCGACTCGTCGATCACTAATGG
CAO-19	GATCACTAATGGACTGTTGTGGTATTATTTTTTTTTGTGCAGTATGCGACTCGTC
CAO-20	GACGAGTCGCATATTACACCCATGTTCGTCA
CAO-21	CCATCCAGCGATTAATCCATTAGTGATC
CAO-22	GGGTATGTTTGGGTAGGGCGGGGACTGTTGTGGTATTATTTTTGGTTGTGCAGT ATGAGGATGA
CAO-23	GGGTATGTTTTCATCCTCCGCCCTACCCAAACATACCCGGAGGATGAGGGTAG GGCGGAGGGTATGTTTGGGTAGGGCGGGTTGGG
CAO-24	AAACATACCCTCCGCCCTACCCTCATCCTCCGGGTATGTTTGGGTAGGGCGGAG GATGAGGGTAGGGCGGAGGATGA
CAO-25	GCTTTCTTCTTTCTTCCCCCCTTGTTTGTTGTTTGC
CAO-26	GGGCTGGGAGGGTTGGGGTATTATTTTTGGTTGTGCCCTATG
CAO-27	ATGGGTCTCACTATGGGACTGTTGTGGTATTATTTTTGGTTGTGCAGTATGACTA
CAO-28	TGGGTAGGGCGGGTTAGTCATCCCATAGTGAGACCCATATGGGTCAAGACATGG GTCTCACTATGGGT
CAO-29	TGGGTAGGGCGGGTAGTGAGACCCATGTCTTGACCCATATGGGATGACTAATGG GTCAAGACATGGGT
CAO-30	TGGGTAGGGCGGGTGTCTTGACCCATTAGTCATCCCATATGGGTCTCACTATGGG ATGACTAATGGGT

## Data Availability

Not applicable.
